# Drug resistance in epilepsy and the *ABCB1* gene: The clinical perspective

**DOI:** 10.4103/0971-6866.80353

**Published:** 2011-05

**Authors:** Abhijit Das, Shabeesh Balan, Moinak Banerjee, Kurupath Radhakrishnan

**Affiliations:** 1 R. Madhavan Nayar Center for Comprehensive Epilepsy Care, Sree Chitra Tirunal Institute for Medical Sciences and Technology, Trivandrum, Kerala, India; 2 Human Molecular Genetics Laboratory, Rajiv Gandhi Center for Biotechnology, Trivandrum, Kerala, India

**Keywords:** *ABCB1*, antiepileptic drugs, drug resistance, epilepsy, genetic association studies, genome-wide association studies, P-glycoprotein, single nucleotide polymorphism

## Abstract

Multidrug resistance is one of the most serious problems in the treatment of epilepsy that is likely to have a complex genetic and acquired basis. Various experimental data support the hypothesis that over-expression of antiepileptic drug (AED) transporters may play a pivotal role in drug resistance. Hyyt 6however, key questions concerning their functionality remain unanswered. The idea that P-glycoprotein, encoded by the *ABCB1* gene, might mediate at least part of the drug resistance was met with both enthusiasm and skepticism. As in oncology, initial optimism has been clouded subsequently by conflicting results. The first study reporting a positive association between genetic variation in the P-glycoprotein and multidrug-resistant epilepsy was published in 2003. Since then, several other genetic association studies have attempted to verify this result. However, taken overall, the role of P-glycoprotein in drug resistance in epilepsy still remains uncertain. We intend to critically review the inherent problems associated with epilepsy pharmacogenetic studies in general and with *ABCB1* polymorphisms studies in particular. The lessons learnt from the *ABCB1* studies can help us to guide future association genetics studies to investigate AED resistance, and thereby taking us closer to the cherished dream of personalized AED therapy.

## Introduction

Epilepsy is the most prevalent chronic neurological disorder and a major public health concern, directly affecting an estimated 50 million people worldwide, and involving an additional 500 million people as family members and caregivers of patients.[1] In a meta-analysis of data obtained from 20 community-based prevalence studies on epilepsy in India, Sridharan and Murthy computed a prevalence rate of 5.3 per 1,000 person-years.[2] A study from Kerala obtained an age-adjusted prevalence rate of 4.7 per 1,000 person-years.[3] On the basis of a prevalence rate of 5 per 1,000 person-years and an incidence rate of 50 per 100,000 person-years, it can be estimated that at any given time, India, with its population of over one billion inhabitants, will have at least 5 million people with active epilepsy, to which nearly 500,000 people will be added to this number every year.

Epilepsies constitute a heterogeneous group of disorders characterized by recurrent unprovoked epileptic seizures due to widely different etiologies. Although a majority of patients with epilepsy are responsive to the presently available antiepileptic drugs (AEDs), nearly one-third of them continue to exhibit recurrent seizures, despite optimal AED therapy.[4,5] Patients with drug-resistant epilepsies are physically and socially disabled, which reduces their quality of life, and are amenable to substantially increased risk of morbidity and mortality.[6,7] Introduction of several new AEDs in the recent years has not improved the outcome of these patients.[7] An enhanced understanding of the underlying mechanisms of AED resistance will help in preventing or reversing resistance.

## An overview of antiepileptic drug resistance

Resistance to AED is considered as a complex phenomenon that may involve many mechanisms, none of which is well understood.[8,9] Any hypothesis(es) concerning the mechanisms of pharmacoresistance should be consistent with the following facts about this condition:[4,10] first, resistance to AED occurs across a wide range of seizure types, etiologies and a variety of AEDs. Second, the majority of patients with drug-resistant epilepsies are unresponsive right from the beginning of AED treatment, indicating that acquired factors are unlikely to explain this phenomenon. Third, the majority of patients who are unresponsive to the first or second AED used continue to remain unresponsive to all AEDs, including the newer ones, and even to multiple AED combinations, indicating that they are multidrug resistant right from the beginning. Fourth, in patients with drug-resistant epilepsies, therapeutic serum AED monitoring and optimizing the dosages seldom results in better seizure control, indicating that decreased AED absorption or increased metabolism is unlikely to be a major cause of AED failure in the majority.

**Figure 1:**
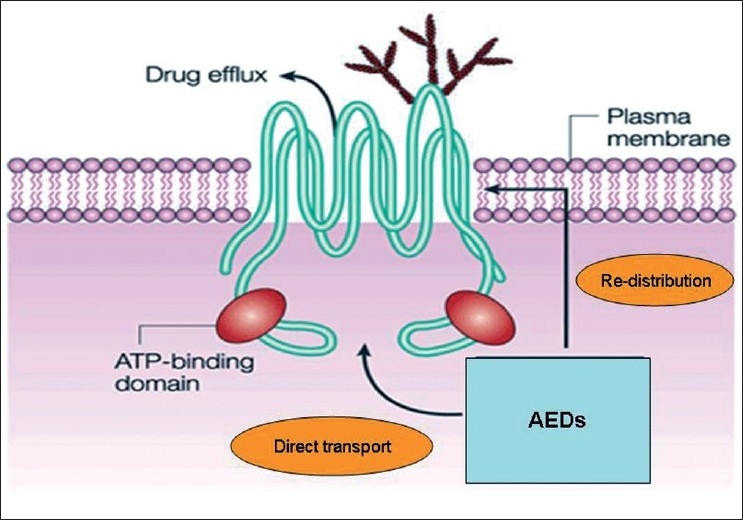
The structure of P-glycoprotein: The structure of P-glycoprotein that transports drugs out of the cell, which is a process that requires the presence of two ATP-binding domains. These domains are a defining characteristic of this family of ATP-binding cassette (ABC) transporters. The exact mechanism of drug efflux is not well understood, but might involve either direct transport out of the cytoplasm or redistribution of the drug as it transverses the plasma membrane. AEDs, anti-epileptic drugs

Currently, two major hypotheses have been put forward to explain AED resistance in epilepsy.[8,9] The *Transporter hypothesis* surmises that region-specific expression or function of mutidrug efflux transporters at the blood-brain barrier [Figure 1] is enhanced, leading to impaired access of AEDs to the central nervous system sites.[11] Consequently, drug concentrations are too low to induce antiepileptic effects at brain sites initiating seizures. The *Target hypothesis* contends that changes in the drug targets (receptors) themselves result in reduced AED sensitivity.[12] Because drug resistance often occurs in a patient to multiple AEDs, if not to all the currently available AEDs simultaneously, the multidrug transporter hypothesis is considered in preference to alterations at specific drug receptor sites to explain the phenomenon of multi-AED resistance. However, the transporter and target hypotheses are not mutually exclusive; they may complement each other in the pathogenesis of AED resistance.

## The role of P-glycoprotein in antiepileptic drug resistance

Numerous multidrug transport proteins are known, and share the general ability to transport a variety of drugs, which often have disparate chemical structures, against concentration gradients, reducing the desired effects of those drugs. P-glycoprotein (permeability glycoprotein, abbreviated as P-gp) is the archetypal example of such a protein, and has been proposed as a mediator of drug resistance and in disparate human conditions, including various cancers, infections such as malaria, inflammatory conditions such as rheumatoid arthritis and Crohn’s disease, and disorders of the central nervous system. P-glycoprotein is a well-characterized ATP-binding cassette (ABC) transporter of the MDR/TAP subfamily and is coded by the *ABCB1* (ATP-binding cassette subfamily B member 1) gene located at the chromosome 7q21.12.[13] P-gp is extensively distributed and expressed in the intestinal epithelium, hepatocytes, renal proximal tubular cells, adrenal gland and capillary endothelial cells comprising the blood–brain and blood–testis barrier. It is an ATP-dependent efflux pump, with a broad substrate specificity, and functions as the major drug–efflux transporter at the blood–brain barrier [Figure 1]. Most AEDs, being planar lipophilic agents, are thus substrates for the *ABCB1* transporter. Because most AEDs are only weak substrates for P-gp, the basal (constitutive) expression of P-gp at the blood–brain barrier is unlikely to restrict brain penetration of AEDs to any clinically important extent.[14] However, intrinsic or acquired over-expression of P-gp in the blood–brain barrier may critically limit the drug penetration into the brain, leading to resistance against all AEDs that are substrates of P-gp.[4,11,14]

To prove the plausibility of a mechanism responsible for drug resistance, the following criteria have been proposed, which remain a good starting point for addressing the issue:[15] (i) resistance mechanisms must be detectable in epileptogenic brain tissue, (ii) resistance mechanisms must have appropriate functionality, (iii) they must be active in drug resistance, and (iv) overcoming the mechanisms should ameliorate drug resistance. Currently, there is extensive evidence that fulfills the above criteria and supports the role of increased expression of P-gp and other drug–efflux transporters in AED-resistance.[4,14] In rodent models of temporal lobe epilepsy, the increased P-gp expression in the hippocampus and the parahippocampal regions was associated with significantly decreased concentrations of AEDs in these regions.[16,17] In patients with oxcarbazepine-resistant epilepsy, the brain tissue expression of *ABCB1* mRNA was found to be inversely correlated with the brain levels of 10,11-dihydro-10-hydroxy-5H-dibenzo(b,f)azepine-5-carboxamide (10-OHCBZ), the active metabolite of oxcarbazepine, indicating that P-gp may play a role in the resistance to oxcarbazepine by causing insufficient concentrations of its active metabolite at neuronal targets.[18] The expression of *ABCB1/MDR1* in the epileptic foci of drug-resistant epilepsy patients was shown to be elevated 10-folds, indicating its significant role in epilepsy.[19] Elevated levels of *MDR1* and multidrug-associated protein (ABCC1/MRP1) have also been associated with pathologies associated with drug refractoriness, such as in tuberous sclerosis, and also in epileptic tissue surrounding dysembryoplastic neuroepithelial tumors, focal cortical dysplasia and hippocampal sclerosis. The adjacent nonepileptic tissue from the same patients did not show elevated expression of the transporter, indicating reduced drug availability to the epileptic foci.[20] Using an *in vitro* blood–brain barrier model with human capillary endothelial cells from either normal brain or drug-resistant epileptic brain, Cucullo *et al*.[21] recently reported a dramatically reduced permeability of phenytoin across the *in vitro* blood–brain barrier formed from endothelial cells of patients with refractory epilepsy, which could be partially counteracted by the selective P-gp inhibitor, tariquidar.[21] In line with this observation, the decrease in brain concentrations and resistance to AEDs, such as phenytoin or phenobarbital, associated with P-gp over-expression in rodent models could be counteracted by tariquidar *in vivo*, suggesting a causal association between P-gp overexpression and AED resistance.[17,22,23] Such P-gp over-expression can result from the effects of disease or drug treatment on P-gp expression or from *ABCB1* polymorphisms, and might explain the clinical observation that patients with refractory epilepsy are usually resistant to a broad range of AEDs with different mechanisms of action.[4]

## *ABCB1* polymorphism in antiepileptic drug-resistance

The human *ABCB1* gene is composed of 29 exons (for details, see latest data releases at *http://www.ensembl.org* and *http://www.ncbi.nlm.nih.gov*). Numerous single nucleotide polymorphisms (SNPs) have been identified in *ABCB1*, half of which reside in coding regions likely to have altered function. The allele frequency for most of the coding region SNPs is low (<8% in different ethnic populations), with the exception of three SNPs in exon 12 (NM_000927.3:c.1236C>T), exon 21 (NM_000927.3: c.2677G>T/A) and exon 26 (NM_000927.3: c.3435C>T). Biological significance of the alleles of these polymorphisms or some of their haplotypes for *ABCB1* activity has been fairly well studied in multidrug resistance. A synonymous SNP in exon 27 (C3435T) was the first variant to be associated with altered protein expression in the human intestinal tract, although the SNP does not change the encoded amino acid.[24] P-gp expression in the duodenum of individuals with the CC genotype was noted to be two-fold higher when compared with that in individuals with the TT genotype, which was associated with significantly decreased plasma concentrations of the P-gp substrate digoxin after oral administration, suggesting lower drug absorption in individuals with high intestinal P-gp levels.[24] The synonymous 3435C>T polymorphism is in linkage disequilibrium with a synonymous SNP in exon 13 (1236C>T) and a nonsynonymous SNP in exon 22 (2677G>TA), suggesting that the observed functional differences in P-gp, initially attributed to the exon 27 synonymous SNP, may be the result of the associated nonsynonymous polymorphism in exon 22, which results in amino acid exchanges (Ala893Ser or Ala893Thr).[25] However, a recent study showed that the synonymous C3435T SNP in exon 27, although not resulting in amino acid changes itself, is not “silent,” but results in P-gp with altered drug and inhibitor interactions.[26]

For the three SNPs described above, ethnic differences have been reported with allele frequencies varying from 45 to 55% in Whites and 5 to 10% in African Americans.[27] The extent of the genotypic effects of these polymorphisms on drug refractoriness in epilepsy has attracted renewed attention.

## Conflicting evidences for the role of *ABCB1* polymorphism in antiepileptic drug resistance

In 2003, Siddiqui *et al*.[28] reported the C3435T polymorphism in the *ABCB1* gene as being associated with resistance to multiple AEDs, and leading to the suggestion, for the first time, that drug resistance in epilepsy might be genetically determined.[28] The study was a retrospective case–control study that compared the frequencies of the *ABCB1* C3435T variant in 115 AED responders, with 200 AED-resistant patients and 200 nonepileptic controls. Patients with multidrug-resistant epilepsy were significantly more likely to be homozygous for the C allele than the T allele. Because the CC genotype has been associated with increased expression of intestinal P-gp,[24] the data of Siddiqui *et al*.[28] suggested that the CC genotype may be associated with increased expression and functionality of P-gp also at the blood–brain barrier, leading to reduced AED levels at their brain targets. In a follow-up study by the same group, the association of AED resistance with the 3435C>T polymorphism was confirmed in a larger group of patients, and intronic sites that are strongly associated with the 3435C>T polymorphism were identified.[29] This study initiated several subsequent genetic association studies, using a candidate gene approach with either one SNP or a haplotype [Table 1]. Six of these studies, genotyping either *ABCB1* 3435 or the common haplotype combination, *ABCB1* 3435C>T-2677G>T-1236C>T, confirmed the association between the 3435 SNP or the three-SNP haplotype (containing the 3435 SNP) and AED-resistant epilepsy. However, in two studies in non-Caucasian subjects, the association was in the reverse direction compared with studies in Caucasian subjects, in that patients with drug-resistant epilepsy were more likely to have the TT genotype compared with those with drug-responsive epilepsy,[31,32] highlighting the complexity of the possible role of *ABCB1* polymorphisms in AED response in different ethnic populations.

**Table 1:** Association studies showing a positive result

Authors	Origin	Polymorphism in *ABCB1*	Number of epilepsy patients		Type of epilepsy/AED	Association of polymorphism with resistance
			Responders	Non responders		
Positive studies						
Siddiqui *et al*.[28] (2003)	UK	3435C>T	115	200	Various/various	Yes
Soranzo *et al*.[29] (2004)	UK	3435C>T	135	286	Various/various	Yes
		IVS 26 + 80T>C			Yes	IVS 26 + 80T>C
Zimprich *et al*.[30] (2004)	Austria	3-SNP haplotypes	—	210	TLE/various	Yes (within the resistant group)
Hung *et al*.[31] (2005)	Taiwan	3-SNP haplotypes	223	108	Various/various	Yes
Seo *et al*.[32] (2006)	Japan	3-SNP haplotypes	84	126	Various/CBZ	Yes (but in reverse direction)
Kwan *et al*.[33] (2007)	China (Hong	3435C>T	297	221	Various/various	Yes (but in reverse direction)
Ebid *et al*.[34] (2007)	Egypt	3435C>T	37	63	Various/PHT	Yes
Hung *et al*.[35] (2007)	Taiwan	3435C>T and 2677G>T	213	114	Various/various	Yes

^a^3-SNP haplotype = 3435C>T, 2677G>T and 1236C>T, ADE = Antiepileptic drug

In contrast to studies showing an association of *ABCB1* polymorphisms and AED resistance, nine other retrospective association studies (including three studies from India) and one prospective cohort study in either Caucasian or non-Caucasian subjects did not identify any significant association between *ABCB1* polymorphism and response to AEDs [Table 2].

**Table 2:** Association studies showing a negative result

Authors	Origin	Polymorphism in *ABCB1*	Number of epilepsy patients		Type of epilepsy/AED	Association of polymorphism with resistance
			Responders	Non responders		
Negative studies						
Tan *et al*.[36] (2004)	Australia	3435C>T	208	401	Various/various	No
Sills *et al*.[37] (2005)	Scotland	3435C>T	170	230	Various/various	No
Kim *et al*.[38] (2006)	Korea	3435C>T	108	63	Various/various	No
Kim *et al*.[39] (2006)	Korea	3-SNP haplotypes	108	99	Various/various	No
Leschziner *et al*.[40] (2006)	UK	3435C>T	503		Various/various	No
Ozgon *et al*.[41] (2007)	Turkey	3435C>T	53	44	Various/CBZ	No
Shahwan *et al*.[42] (2007)	Ireland	3435C>T and other SNPs	242	198	Various/various	No
Lakhan *et al*.[43] (2008)	India	3435C>T and other SNPs	231	94	Various/various	No
Vahab *et al*.[29] 2009	India	3435C>T and other SNPs	129	113	Various/various	No
Grover *et al*.[44] 2010	India	3435C>T and other SNPs	95	133	Various/various	No

^a^3-SNP haplotype = 3435C>T, 2677G>T and 1236C>T, ADE = Antiepileptic drug

One recent meta-analysis failed to find any association between *ABCB1* genotype and response to anticonvulsant drugs [odds ratio 1.15; 95% confidence interval (CI) 0.78–1.70; *P* = 0.48)]. Subanalysis of studies according to ethnicity yielded similar findings [European cohort: OR = 1.31; 95% CI 0.89-1.94, *P* = 0.18; Asian cohort: OR = 0.99; 95% CI 0.51-1.89, *P* = 0.96)].[45]

## Lack of replication: The causes and their clinical implications

All the studies done so far in *ABCB1* polymorphisms and AED-resistance in epilepsy are candidate gene-based genetic association studies, either SNP based or haplotype based, with their inherent limitations. Risch and Merikangas[46] identified SNPs as putative genetic risk factors for association testing and proposed a genome wide-significance level set at the very low value of 10^-8^ to allow for the total number of intragenic SNPs in the human genome. Because most current studies are underpowered to achieve such a stringent level of significance, replications are usually necessary for the confirmation of an association finding. Hirschhorn *et al*.[47] conducted a meta-analysis of 166 initial association findings and their subsequent attempted replications for a large number of complex disorders. They included putative association findings for which at least two subsequent replication attempts have been published, and they found that only six of the 166 initial findings have been reliably replicated (with >75% of replication studies showing significant results). Similar surveys of the association literature have been conducted, yielding successful replication rates of 16–30%.[48,49] Hence, the failure of replication is not unexpected in studies of pharmacoresistance in epilepsy, which is a heterogeneous and complex problem. In addition to inconsistent phenotype definition among studies, which is the most critical issue in the problem of nonreplication, there are various other potential explanations for the discordant results, including inadequate power, potential confounding by comorbidity and comedication, population substructure, genotyping error, overlap in substrate specificity between P-gp and other drug efflux transporters and inclusion of AEDs that might not be P-gp substrates.[50]

In this review, we have limited ourselves to the issues that are more important from the clinical perspective. The clinician should be aware of these issues for a better understanding of the genetic association studies and to make valuable contributions in the future studies.

## Phenotypic heterogeneity

Epilepsy is a heterogeneous condition. Different phenotypes not only indicate different pathomechanisms and drug responsiveness but also a difference in the underlying epigenetic mechanisms. In the original study, Siddque *et al*.[28] defined drug resistance as the occurrence of at least four seizures over the year before recruitment with trials of more than three appropriate AEDs at maximal tolerated doses, which were established on the basis of the occurrence of clinical side-effects at supramaximal doses. Where it was possible, only the recognized, more specific drugs for a syndrome were considered appropriate – e.g., failure of a case of idiopathic generalized epilepsy to respond to carbamazepine was not considered a failure for the purposes of the study. However, only in one of the subsequent studies[36] was the above definition for drug resistance replicated. There is no consistent clinical definition of multidrug resistance in epilepsy, and agreement among the different definitions is strong but imperfect.[51] This has resulted in diverse criteria used by different researchers, or even a lack of explicit criteria in some cases, rendering it difficult to compare the findings across studies. The recent consensus definition of drug resistance, as provided by the Task Force appointed by the International League Against Epilepsy (ILAE), is a much needed step to address this issue.[52] However, it should be emphasized that given the paucity of high-quality data on the long-term prognosis of epilepsy, the proposed definition should not be regarded as a foregone conclusion, but is intended to represent a consensus opinion that needs to be tested in rigorous prospective studies and refined as new evidence emerges.

As patients with multidrug resistance will typically not respond to a variety of AEDs with differing modes of action, it has been suggested that the biological mechanism underlying multidrug resistance is nonspecific, i.e. it is not unique to any one particular AED or subset of AEDs. However, this notion has been recently challenged by the elegant study done by Kimchi-Sarfaty *et al*.,[26] who showed that a synonymous SNP (i.e., a so-called silent mutation) at the 3435 locus of the *ABCB1* gene alters P-gp conformation and its interaction with drug substrates and inhibitors, while expression levels of mRNA and protein remain the same. The synonymous mutation apparently affects the timing of cotranslational folding of P-gp. Hence, the transport function of P-gp not only depends on genotype at specified loci within *ABCB1* but also, critically, that differences in transport function are exaggerated when the transcription machinery is put under stress. Thus, the genotype dependence of transport function is exaggerated under the stimulated state in comparison with the basal or repressed state. In specific experimental models, both seizures and AED treatment can induce *ABCB1* expression, and would therefore be expected to amplify any *ABCB1* genotype effect on P-gp quantity and function.[11] Therefore, if phenotypes are not matched between studies, e.g. if one study focuses on a cohort with a more severe “drug-resistant” phenotype (in comparison with a control cohort) while another study examines a less-severe “drug-resistant” cohort, there is good reason to believe that genotype-dependent differences should be expected between these studies. Thus, a stratification bias would already exist and misdirect the results toward nonreplication from the outset.[53] This point duly emphasizes the importance of matching phenotypes across studies or cohorts.

## Are all antiepileptics transported by P-gp?

Fifteen of the 18 genetic association studies summarized in Tables 1 and 2 included patients on treatment with various AEDs, for several of which it is either not yet known whether they are transported by P-gp or which do not seem to be transported by P-gp (e.g., valproate).[54] Only three studies included patients on a single AED, either phenytoin or carbamazepine.[32,34,41] For carbamazepine, data on transport by P-gp are, at best, equivocal,[55,56] whereas there is ample evidence that phenytoin is transported by P-gp,[57] which could explain the recent finding of Ebid *et al*.[34] that the CC genotype of *ABCB1* 3435 is significantly associated with phenytoin resistance in patients with epilepsy. It should be noted that the results of the Ebid *et al*.[34] study differed substantially from other similar studies, in that a highly significant association between 3435CC and resistance to phenytoin was found despite a smaller sample size (100 patients only compared with >300 in most other studies). A number of other concerns exist about the clarity of the reports and the study designs. Hence, the studies containing patients who use a set of AEDs different from another patient cohort are not directly comparable. It is also important to remember the discussion made previously, in light of the findings by Kimchi-Sarfaty *et al*.,[26] that P-gp functionality will also depend on the AEDs used, making the comparison much more complicated. As the multidrug-resistant patients will continue to use many AEDs, often differing along the course of illness, it would be exceedingly difficult, if not impossible, to control this important confounding factor.

## Is P-gp the only antiepileptic exporter?

Almost all previous studies on gene variation that might affect AED distribution have dealt with polymorphisms in the *ABCB1* gene that encodes P-gp. However, there are polymorphisms in other drug efflux transporters, such as members of the MRP family, which may affect the distribution of AEDs[57] and need further investigation. One of these transporters, RLIP76, has been suggested to be involved in AED resistance by transporting both carbamazepine and phenytoin at the blood–brain barrier,[58] but a recent genetic analysis of RLIP76 genotypic and haplotypic frequencies in 783 patients with epilepsy and 359 healthy controls showed no significant differences for genotypic frequencies between drug-resistant and drug-responsive patients.[59]

## Is *ABCB1* associated with epilepsy *per se*?

Several of the many association studies on *ABCB1* in epilepsy not only analyzed drug-responsive and -nonresponsive epilepsy patients but also analyzed the *ABCB1* genotypes in control subjects without epilepsy, and found that in fact *ABCB1* genotypes were associated with the disease per se rather than specifically with drug responsiveness.[14] In our study from patients with south Indian ancestry (unpublished), we also had similar findings but with a different SNP from the *ABCB1* gene. This suggests that the reported association studies on this gene may be looking simply at the random segregation of epilepsy patients into the two drug-response groups, such that in half of the studies an association was found and in the other half of the studies the results were negative.

## The question of power

The most fundamental problem with current efforts is the relatively low sample size in most studies, resulting in limited power to either detect or to rule out a definitive association. Few investigators working on epilepsy genetics have large cohorts for any particular type of epilepsy, including the recent and previous efforts. It does not appear that, in the near future, individual investigators will be able to increase their own sample sizes to levels sufficient to carry out highly powered association studies within the various forms of epilepsy.[60] The following pertinent lessons could be learned from the lack of consistency in the results from different studies on *ABCB1* polymorphisms on drug resistance: first, the field must concentrate more seriously on efforts to determine which polymorphisms have real effects, as opposed to always racing to publish a new association. False-positives are clearly exacerbated by the publication of multiple small studies and the practice of data exploration to identify subgroups that show associations. This fact must be recognized and addressed. Second, and most fundamental, it is critical for different research groups to increase substantially the size of their patient cohorts. Given that this will take time, we feel that in the short term, groups should combine their epilepsy samples and attempt to replicate one another’s results in a population of similar ethnicity before publication. Although collaboration to such a level could be viewed as controversial from a scientific point of view, we feel that the trade-off is justified. It is only with such steps that the epilepsy community, like other disease subspecialties, will be able to arrive at a reasonable false to true discovery ratio in reported associations.

## Genome-wide association studies: Is it the future?

The techniques of a candidate gene-based approach are limited because our current understanding of gene function is too limited to allow us to predict which genes are involved with a particular trait. The genome wide association studies (GWAS), which agnostically evaluate the patterns of association between variation in the genome and carefully defined disease phenotypes, present an enhanced experimental framework to rapidly identify novel pathogenic mechanisms and to confirm a role for previously known mechanisms.[61] The completion of the human genome sequence in 2005 and the provision of an initial catalogue of human genetic variation and a haplotype map (known as the HapMap), together with rapid improvements in genotyping technology and analysis, have permitted genome wide association studies to be undertaken in a large number of samples.[62] In the first and current implementation of this approach, the great majority of genetic variants with population frequencies of 5% or more could be tested directly or indirectly for association with disease risk or quantitative traits thus providing a potential path to gene discovery for polygenic diseases and traits. During the past couple of years, GWAS have identified more than 250 genetic loci, in which common genetic variants occur that are reproducibly associated with polygenic traits.[63] This explosion represents one of the most prolific periods of discovery in human genetics. However, the problems of phenotypic assertion and population stratification will also continue to plague this technique, exaggerated by the inherent complicated issue of multiple testing in GWAS. GWAS simply escalates the problem of multiple testing by orders of magnitude. Because SNP-based GWAS test hundreds of thousands of SNPs per subject, a significant association requires a very low probability value.[64] The Wellcome Trust Case Control Consortium used *P* < 5×10^-7^ as the cut-off for genome wide significance. Others have chosen to prespecify genome wide significance with greater stringency at *P* < 5×10^-8^, corresponding to the 5% significance level adjusting for the number of independent tests estimated in HapMap for individuals of European ancestry.[64] This leads to the requirement of a large sample size, which is already a problem in epilepsy genetics. Altshuler and Daly[65] calculated sample sizes required to have 90% power to repeat the findings of previous GWAS at a *P*-value threshold of 10^-8^. To put things in perspective, the most strongly associated SNPs would individually require 2,500 cases and controls; most would need 10,000–20,000. In many of the diseases studied so far, there are multiple SNP associations such that, although 20,000 cases may be required to guarantee identifying a given SNP, smaller studies may still identify a proportion of SNP associations. Recent large GWAS have provided empirical justification for stringent *P*-values. A recently reported, two-stage GWAS in breast cancer included over 25,000 cases and 25,000 controls.[66] In the first stage, 4,000 cases and controls were genotyped. The most significant SNPs were retested in an independent sample of 21,000 cases and controls. This large study offers insight into the relationship between *P*-values in stage 1 and confirmation in stage 2. No SNPs with a *P*-value less stringent than 10^-6^ survived stage 2 and *P*-values between 10^-7^ and 10^-8^ had some chance of failure. Hence, while it has been argued that *P*-values as high as 10^-5^ may be sufficient in stage 1, there is increasing consensus that *P*-values of 10^-8^ are required.

There is what might be called large numbers of imperatives when it comes to GWAS of a complex disorder like epilepsy. It is unlikely that any single study, even a multicenter study, will ever achieve the sample size necessary to yield a credible result. An uncommon level of worldwide collaboration must emerge, which has already started in some other neurological disorders.[67]

## Conclusions

The role of genetic variation in drug efflux transporter genes for AED distribution and efficacy remains uncertain at present. Progress in the pharmacogenomics of multidrug resistance in epilepsy has been slow. However, a number of lessons can be learnt from the studies conducted. A clear definition of the multidrug-resistant phenotype is fundamental in order to ensure consistency among studies and to ensure that patients classified as multidrug resistant are indeed unlikely to respond to any AED. Heterogeneity of AED treatment among patients also needs to be considered, e.g. if overexpression of P-gp is a factor in multidrug resistance, then a different mechanism would be required to explain nonresponse to an AED that is not thought to be a substrate for P-gp (e.g., vigabatrin). Functional studies must support association genetics to establish biological plausibility. Investigating multidrug resistance for certain epilepsy subtypes or AED combinations will be a difficult task. Association genetics requires large cohort sizes, and further refining the multidrug-resistant phenotype or inclusion criteria will make it harder to recruit sufficient patients. GWAS will require particularly large cohorts given the number of polymorphisms examined and the number of likely false-positive associations. Collaboration between centers will prove increasingly important not only to increase cohort sizes but also to replicate genetic associations.
